# Generation of a Cell Culture-Adapted Hepatitis C Virus with Longer Half Life at Physiological Temperature

**DOI:** 10.1371/journal.pone.0022808

**Published:** 2011-08-04

**Authors:** Chon Saeng Kim, Sun Ju Keum, Sung Key Jang

**Affiliations:** 1 Molecular Virology Laboratory, POSTECH Biotech Center, Department of Life Science, Pohang University of Science and Technology, Pohang, Kyungbuk, Republic of Korea; 2 Division of Integrative Biosciences and Biotechnology, Pohang University of Science and Technology, Pohang, Kyungbuk, Republic of Korea; 3 Biotechnology Research Center, Pohang University of Science and Technology, Pohang, Kyungbuk, Republic of Korea; Institut Pasteur Korea, Korea, Republic of

## Abstract

**Background:**

We previously reported infectious HCV clones that contain the convenient reporters, green fluorescent protein (GFP) and *Renilla* luciferase (Rluc), in the NS5a-coding sequence. Although these viruses were useful in monitoring viral proliferation and screening of anti-HCV drugs, the infectivity and yield of the viruses were low.

**Methodology/Principal Findings:**

In order to obtain a highly efficient HCV cultivation system, we transfected Huh7.5.1 cells [1] with JFH 5a-GFP RNA and then cultivated cells for 20 days. We found a highly infectious HCV clone containing two cell culture-adapted mutations. Two cell culture-adapted mutations which were responsible for the increased viral infectivity were located in E2 and p7 protein coding regions. The viral titer of the variant was ∼100-fold higher than that of the parental virus. The mutation in the E2 protein increased the viability of virus at 37°C by acquiring prolonged interaction capability with a HCV receptor CD81. The wild-type and p7-mutated virus had a half-life of ∼2.5 to 3 hours at 37°C. In contrast, the half-life of viruses, which contained E2 mutation singly and combination with the p7 mutation, was 5 to 6 hours at 37°C. The mutation in the p7 protein, either singly or in combination with the E2 mutation, enhanced infectious virus production about 10–50-fold by facilitating an early step of virion production.

**Conclusion/Significance:**

The mutation in the E2 protein generated by the culture system increases virion viability at 37°C. The adaptive mutation in the p7 protein facilitates an earlier stage of virus production, such as virus assembly and/or morphogenesis. These reporter-containing HCV viruses harboring adaptive mutations are useful in investigations of the viral life cycle and for developing anti-viral agents against HCV.

## Introduction

Over 180 million people worldwide are chronically infected with hepatitis C virus (HCV), and are thus at high risk of developing chronic liver diseases such as progressive hepatic fibrosis, liver cirrhosis and hepatocellular carcinoma [2], [3]. No HCV vaccine is available to date, and there is no therapy that is effective for all genotypes of HCV. Interferon-alpha (IFN-α) in combination with ribavirin is the only recommended therapy [4]. These treatments have a moderate response rate and are associated with significant side effects [5], [6].

HCV is a member of the *Flaviviridae* family of enveloped, positive-strand RNA viruses [7]. The HCV genome consists of an approximately 9.6-kb RNA molecule containing a large open reading frame flanked by 5′ and 3′ non-translated regions (5′ and 3′NTRs). The viral proteins are translated as a single large polyprotein of 3,010–3,040 amino acids, which is co- and/or post-translationally processed by cellular and viral proteases into mature structural (core, E1, E2) and nonstructural (p7, NS2, NS3, NS4a, NS4b, NS5a, NS5b) viral proteins [8]. The envelope proteins, E1 and E2, are extensively glycosylated and form a non-covalent complex that is believed to represent the building block for the viral envelope [9], [10]. P7 is a 63-amino-acid polypeptide that is often incompletely cleaved from E2. It has two transmembrane domains connected by a short hydrophilic segment [11]. The p7 protein probably forms an ion channel involved in some step of virus production [12]. *In vivo* experiments clearly indicate that p7 is essential for infection, and two recent reports showed that p7 is essential for production of infectious virions [13], [14], [15].

The availability of a cell culture system is a prerequisite for studying the entire life cycle of a virus and to devise strategies for prophylactic and therapeutic interventions [16]. The most recent advance in this context is the development of a virus production system based on the transfection of the human hepatoma cell line, Huh7.5.1, with genomic HCV RNA (JFH1) isolated from a patient with fulminant hepatitis [1], [17], [18], [19].

Previously, we reported infectious HCV clones that contain the convenient reporters, green fluorescent protein (GFP) and *Renilla* luciferase (Rluc), in the NS5a-coding sequence [20]. Although these viruses were useful for monitoring the effects of antiviral agents and for studying viral replication cycles, their infectivity was too low for mass production of HCV virions. Here, we report cell culture-adapted, reporter-containing HCV clones. A T563I mutation in the E2 coding region increased the viability of infectious virus in culture media at 37°C, and an N765D mutation in the p7 protein increased virus production by augmenting an early step of virion production. These cell culture-adapted infectious viruses will facilitate HCV-related research, including the development of anti-HCV drugs and vaccines.

## Results

### Generation of cell culture-adapted JFH 5a-GFP virus

**Figure 1:**
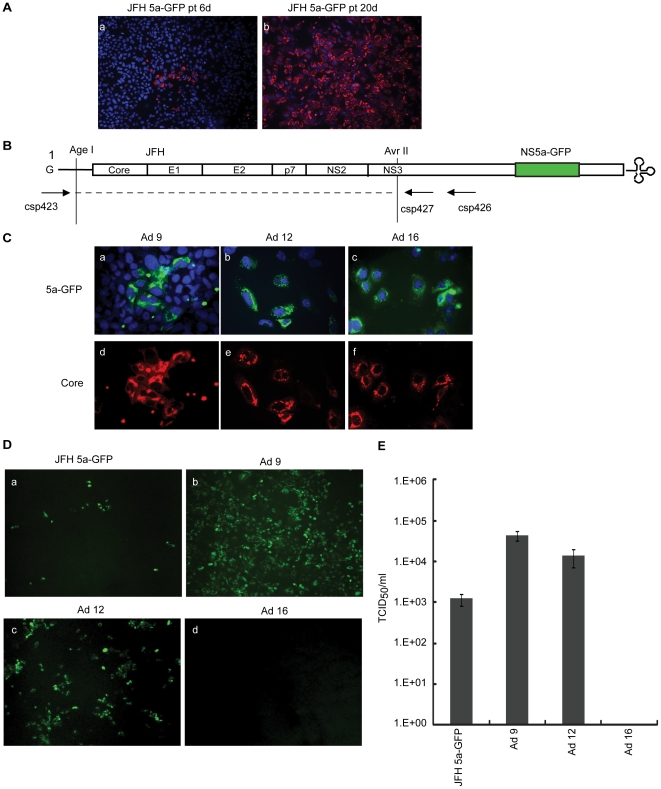
Generation of cell culture-adapted JFH 5a-GFP virus. (A) Cell-free culture supernatants were collected 6 days (JFH 5a-GFP pt6d) and 20 days (JFH 5a-GFP pt20d) after transfection, and used to inoculate Huh7.5.1 cells. HCV-infected cells were fixed 5 days after inoculation and then treated with primary (anti-core monoclonal) and secondary (Alexa 555-conjugated donkey anti-mouse IgG) antibodies. The core-expressing cells are shown in red and the Hoechst 33258-stained nuclei are shown in blue. (B) Schematic diagram of JFH 5a-GFP and its derivatives with a substituted region (from Age I site to Avr II site) containing amplified DNA fragments from long distance RT-PCR of cell-adapted HCV RNA. (C) Huh7.5.1 cells transfected with JFH 5a-GFP, Ad 9, Ad 12 or Ad 16 RNAs were grown on coverslips for 3 days and fixed. Core-expressing cells are shown in red as in 1A (panels d to f). The NS5a-GFP signal was visualized by fluorescence microscopy (green). The nuclei, shown in blue, were stained with Hoechst 33258 (panels a to c). (D) Huh7.5.1 cells were infected with JFH 5a-GFP, Ad 9, Ad 12 or Ad 16 viruses. The NS5a-GFP signals were visualized by fluorescence microscopy (green). (E) Virus titers were determined using a TCID_50_ assay. The bars and lines represent the means and standard deviations, respectively, from three independent experiments.

As a first step to obtaining a highly efficient HCV cultivation system, we transfected Huh7.5.1 cells with JFH 5a-GFP RNA [20] and then cultivated cells for 20 days. Culture supernatants harvested 6 and 20 days after transfection were used to inoculate Huh7.5.1 cells, and the expression of core protein in infected cells was examined by immunocytochemistry at 5 days post-infection. As shown in Figure 1A, nearly 100% of cells from the media obtained 20 days after transfection were infected. In contrast, only a few cells from the media obtained 6 days after transfection were infected. This difference in infectivity between 6-day and 20-day post-transfection media could be an indication that infectious virions containing adaptive mutations accumulated over time in the media. This possibility was tested by isolating and analyzing individual virus clones obtained from cells infected with the media obtained 20 days after transfection. To identify cell culture-adaptive mutations, we prepared total RNA from infected cells and amplified the region encoding the core to NS2 by long distance RT-PCR, as described in Materials and Methods (Fig. 1B). After restriction enzyme digestion of PCR products with AgeI and AvrII, this DNA fragment was inserted into the parental JFH 5a-GFP construct (Fig. 1B). *In vitro* transcripts of 12 independent clones were prepared and transfected into Huh7.5.1 cells to investigate the expression of core and NS5a-GFP protein. As shown in Figure 1C, three clones, designated Ad 9, Ad 12 and Ad 16 expressed the core and NS5a-GFP protein. The remaining nine clones did not express the core or NS5a-GFP protein. Cell culture supernatants obtained 5 days after transfection with Ad 9, Ad 12 and Ad 16 RNAs were examined for infectious virus production by fluorescence microscopy and tested in a TCID_50_ assay. Huh7.5.1 cells inoculated with culture supernatants were monitored by fluorescence microscope at 5 days post-infection (Fig. 1D). Ad 9 and Ad 12 viruses showed higher infectivity compared with the JFH 5a-GFP virus (panels a, b and c in Fig. 1D), whereas cells inoculated with Ad 16 supernatant exhibited no NS5a-GFP expression (panel d in Fig. 1D). The viral titers of Ad 9 and Ad 12, measured by a TCID_50_ assay, were 36-and 11-fold higher, respectively, than parental JFH 5a-GFP (Fig. 1E). These results suggest that adaptive mutations in Ad 9 and Ad 12 constructs increased virus infectivity. The absence of infectivity in the Ad 16 construct, despite viral RNA replication and the production of viral protein, is consistent with the presence of an additional mutation in the Ad 16 construct that abolished virus infectivity.

### Mutations in E2 and p7 increase viral infectivity

**Figure 2:**
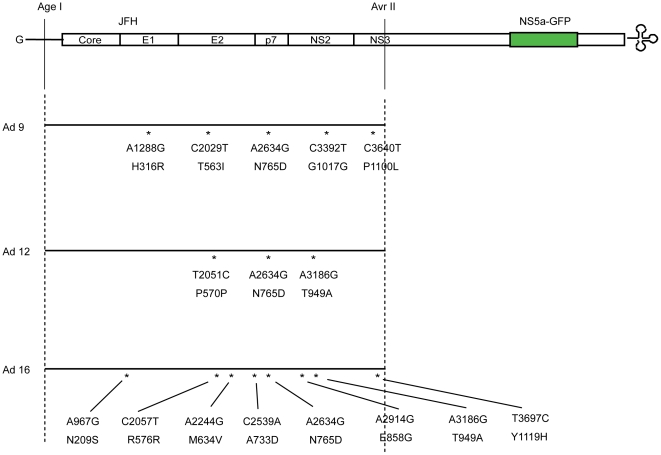
Genetic mutations identified in cell culture-adapted HCV RNAs. A schematic diagram of JFH 5a-GFP is shown at top. The positions of predicted amino acid changes are depicted in bold characters, and those with silent mutations are depicted in plain characters.

To identify the mutations responsible for enhanced virus infectivity, we sequenced the entire replaced regions of the Ad 9, Ad 12 and Ad 16 constructs (Fig. 2). Five mutations were identified in the Ad 9 construct, one of which was silent (C→T at nucleotide position 3392). Four led to changes in amino acids: His 316 to Arg, Thr 561 to Ile, Asn 765 to Asp, and Pro 1100 to Leu. Three and eight mutations were identified in Ad 12 and Ad 16 constructs, respectively. Interestingly, the N765D mutation (in p7) was identified in all three constructs.

**Figure 3:**
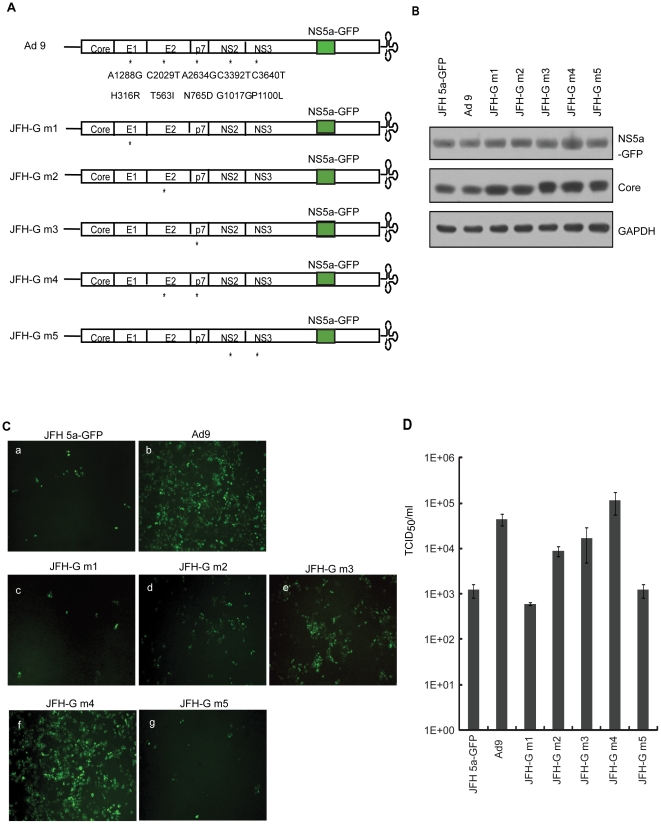
Mutations in E2 and p7 proteins increased viral infectivity. (A) Schematic diagram of JFH 5a-GFP and its derivatives containing adaptive mutations. Mutations are indicated with an asterisk; dedicated names are on the left. (B) Western blot analysis of Huh7.5.1 cells transfected with JFH 5a-GFP or its derivative RNAs containing adaptive mutations. Protein levels were analyzed by western blotting with anti-NS5a, anti-core or anti-GAPDH antibodies, respectively. (C) Huh7.5.1 cells were infected with JFH 5a-GFP, Ad 9, JFH-G m1, JFH-G m2, JFH-G m3, JFH-G m4 or JFH-G m5 viruses, and the NS5a-GFP signal was visualized by fluorescence microscopy (green). (D) Viral titers were determined using a TCID_50_ assay. The bars and lines represent the means and standard deviations, respectively, from three independent experiments.

To determine which of these mutations was responsible for the increased viral infectivity, we re-introduced the mutations identified in the most efficient clone (Ad 9), alone and in combination into the JFH 5a-GFP construct, to yield JFH-G m1 (mutation in E1 only), JFH-G m2 (mutation in E2 only), JFH-G m3 (mutation in p7 only), JFH-G m4 (E2+p7 mutations) and JFH-G m5 (NS2+NS3 mutations) (Fig. 3A). First, we compared viral protein expression in cells transfected with the parental JFH 5a-GFP with that in cells transfected with its derivatives containing adaptive mutations (Fig. 3B). RNAs transcribed *in vitro* were transfected into Huh7.5.1 cells by electroporation. Three days after transfection, cell lysates were prepared and the levels of NS5a-GFP and core protein were investigated by western blot analysis using anti-NS5a and anti-core antibodies (Fig. 3B). Similar levels of core and NS5a-GFP proteins were observed in cells transfected with all RNAs. The infectivity of JFH 5a-GFP RNA and its derivative RNAs (Ad 9, JFH-G m1, JFH-G m2, JFH-G m3, JFH-G m4 and JFH-G m5) was monitored by inoculating Huh7.5.1 cells with culture media from Huh7.5.1 cells transfected with the corresponding RNAs and cultivated for 5 days. At 5 days post-infection, NS5a-GFP fluorescence was observed by fluorescence microscopy (Fig. 3C). The combination of E2 and p7 mutations in JFH-G m4 increased viral infectivity (compare panels f and a in Fig. 3C); none of the mutations in JFH-G m1 or JFH-G m5 viruses had a significant effect (panels c and g in Fig. 3C). Although the mutations in E2 and p7 proteins (T563I and N765D) both increased virus infectivity, the patterns within infected cells were different for each, with the E2 mutation (JFH-G m2) exhibiting a dispersed intracellular distribution and p7 (JFH-G m3) showing infection of cells at focal regions (compare panels d and e in Fig. 3C).

An analysis of virus titer in culture supernatants yielded similar results (Fig. 3D). The viral titer of the JFH-G m4 virus (containing both mutations) was ∼100-fold higher than that of the JFH 5a-GFP virus (Fig. 3D), whereas that of JFH-G m2 and JFH-G m3 containing individual mutations in E2 and p7, respectively, each increased by approximately 10-fold (Fig. 3D). The reporterless viruses with the corresponding mutations showed similar virus production to the mutant viruses with the reporter gene (Fig. S1). In contrast, the titer of the JFH-G m1 virus was reduced about 50% compared with that of JFH 5a-GFP virus, indicating that the mutation in E1 protein (H316R) had a slightly negative effect on virus infectivity (compare columns JFH-G m1 and JFH 5a-GFP in Fig. 3D). The lower infectivity of Ad 9 relative to JFH-G m4 may be attributable to the opposing influence on infectivity caused by the presence of the E1 protein mutation in Ad 9 (compare columns Ad 9 and JFH-G m4 in Fig. 3D). The mutations in NS2 and NS3 proteins had no effect on viral infectivity (compare columns JFH-G m5 and JFH 5a-GFP in Fig. 3D). Taken together, these results suggest that two mutations, one in E2 and one in p7, enhance viral infectivity.

### The E2 mutation increases virion viability and the p7 mutation augments virion assembly

**Figure 4:**
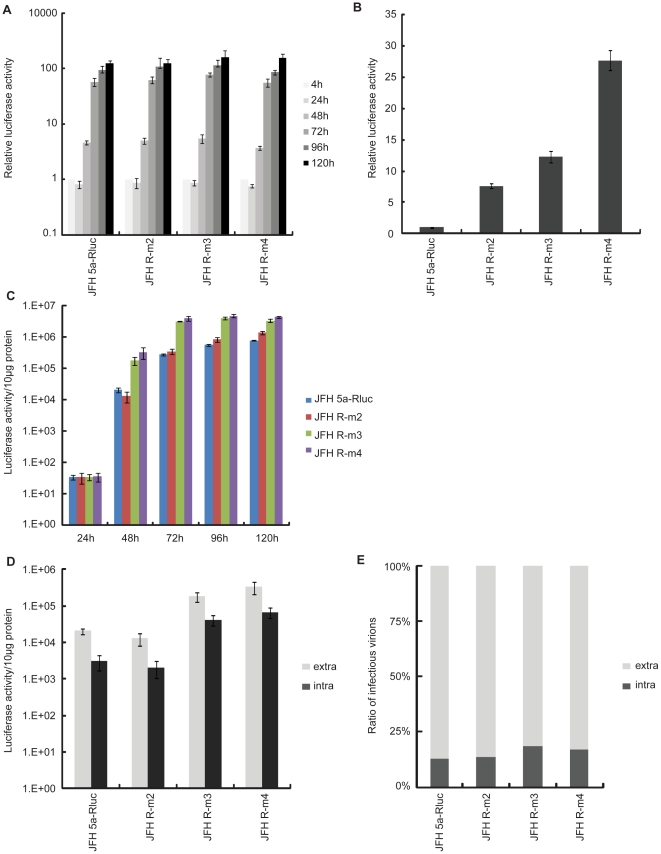
A mutation in the p7 protein augmented a virion assembly step. (A) Replication of JFH 5a-Rluc, JFH-R m2, JFH-R m3 and JFH-R m4 RNAs was investigated using a luciferase reporter assay. Cells were transfected with RNAs and harvested at the indicated times. Luciferase activity values are expressed relative to reporter activity measured at 4 hours (reference value = 1). The bars and lines represent the means and standard deviations, respectively, from three independent experiments. (B) Infectivity of culture fluids harvested 5 days after transfection with JFH 5a-Rluc, JFH-R m2, JFH-R m3 or JFH-R m4 RNAs was determined by measuring luciferase activity in cells 3 days after inoculation of Huh7.5.1 cells. Luciferase activity values are expressed relative to reporter activity measured in cells infected with the JFH 5a-Rluc virus (reference value = 1). The bars and lines represent the means and standard deviations, respectively, from three independent experiments. (C) The kinetics of virus release into the media of transfected cells in panel A were measured. Culture supernatants were harvested at the indicated times and used to inoculate Huh7.5.1 cells. Three days after infection, luciferase activity in cells was measured and normalized to protein concentration, determined by the Bradford assay. The bars and lines represent the means and standard deviations, respectively, from three independent experiments. (D) Two days after transfection, culture media were harvested to collect extracellular virus. Virus-producing cells were washed and lysed by four freeze-thaw cycles to release intracellular virus. Infectious virus titers in the media and inside the cells were investigated by infecting Huh7.5.1 cells with media and cell lysates, respectively. The extracellular (white bars) and intracellular (gray bars) viral titers were measured by luciferase assay. The relative ratios of intracellular and extracellular infectious viruses are shown in panel (E). The bars and lines represent the means and standard deviations, respectively, from three independent experiments.

Because it is easier to quantify luciferase activity than GFP signals, we constructed JFH 5a-Rluc viruses containing either mutation (JFH-R m2: E2 mutation; JFH-R m3: p7 mutation) and both mutations (JFH-R m4) to elucidate the roles of adaptive mutations in E2 and p7 protein. Viral protein production reflecting RNA replication and translation efficiencies of the adaptive mutants was determined by measuring luciferase activity in transfected cells for 5 days (Fig. 4A). Luciferase activity assays showed that there were no significant differences in RNA replication or translation among cells transfected for 120 hours with RNAs for JFH 5a-Rluc, JFH-R m2, JFH-R m3 or JFH-R m4 viruses (Fig. 4A). To determine the infectivity of these viruses, we inoculated Huh7.5.1 cells with culture supernatants obtained 5 days post-transfection and measured luciferase activity in the cells. Consistently with the results obtained using JFH 5a-GFP constructs of viruses with adaptive mutations (Fig. 3D), JFH 5a-Rluc virus constructs containing individual mutations in E2 and p7 protein each increased infectivity by ∼10-fold, and the JFH-R m4 virus containing both mutations showed the highest infectivity (Fig. 4B). These results indicate that the two adaptive mutations are each responsible for a similar increase the infectivity of JFH 5a-Rluc and JFH 5a-GFP viruses.

To monitor viral release at various times after transfection, we inoculated Huh7.5.1 cells with culture media harvested 24, 48, 72, 96 and 120 hours after transfecting with each RNA, and then cultivated cells for 3 days. Luciferase activity in the HCV-infected cells is shown in Figure 4C. The mutation in the p7 protein, either singly or in combination with the E2 mutation, enhanced virus release by ∼10–50-fold (compare columns in Fig. 4C). This effect was more pronounced at early time points (48 and 72 hours) than at the last time point (120 hours). The mutation in the E2 protein had a minor impact on the release of infectious virions (Fig. 4C). To further investigate which step of virion production was enhanced by the adaptive mutation in the p7 protein, we compared the infectivity of intracellular virus to that of extracellular virus. At 48 hours post-transfection, culture media were harvested to collect the extracellular virus and cells were washed and lysed by four freeze-thaw cycles to release intracellular virus. Huh7.5.1 cells were infected with the cell lysates or culture media, and then luciferase activity in the infected cells was measured 3 days after infection. The infectivity of extracellular virus (white column) and that of intracellular virus (gray column) is shown in Figure 4D. The ratios of intracellular viruses to extracellular viruses obtained from various mutants are shown in Figure 4E. The number of infectious virions in both culture media and inside cells was ∼10-fold higher with RNAs containing the p7 mutation (compare columns JFH-R m3 and JFH-R m4 with columns JFH 5a-Rluc and JFH-R m2 in Fig. 4D), consistent with the result shown in Figure 4C. Importantly, the ratios of intracellular to extracellular infectious virions were approximately the same for all viruses (Fig. 4E), indicating that the p7 mutation does not affect viral release. Collectively, the results presented in Figure 4 support the conclusion that the adaptive mutation in the p7 protein facilitates an earlier stage of virus production, such as virus assembly and/or morphogenesis.

**Figure 5:**
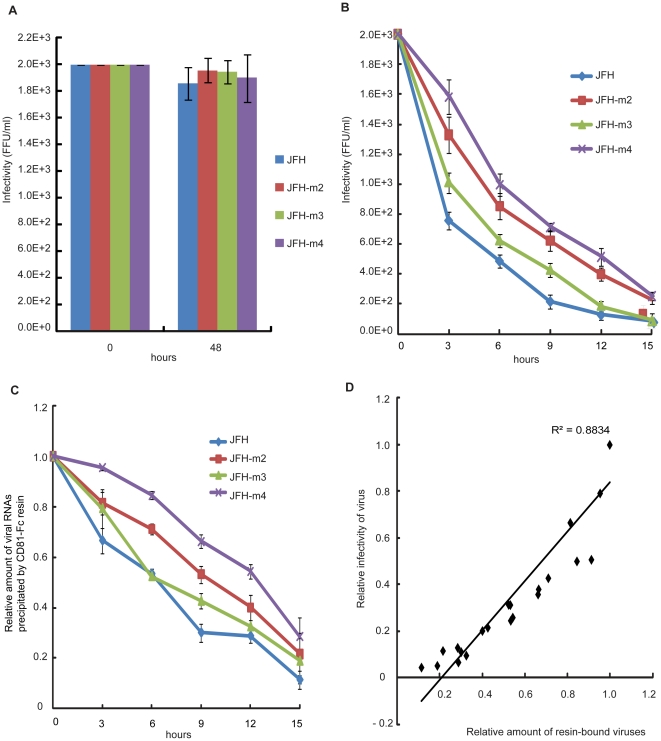
A mutation in the E2 protein increased virion viability at 37°C. (A and B) Infectious viral titers remaining in the media were determined by infecting Huh-7.5.1cells after same amount of JFH, JFH-m2, JFH-m3 and JFH-m4 viruses were incubated at 4°C (A) or 37°C (B) for the indicated times. Viral titers were determined by counting numbers of plaques visualized by an immune-fluorescence method. (C) The same infectious dose of JFH, JFH-m2, JFH-m3, and JFH-m4 viruses were incubated at 37°C for the indicated times, and then CD81-interacting viruses were precipitated by a protein G agarose resin conjugated with CD81-Fc fusion protein. The amounts of resin-bound virions were measured by quantitative RT-PCR. The relative amount of resin-bound viruses is depicted. The dots and lines represent the means and standard deviations, respectively, from three independent experiments. (D) The relationship between CD81-binding capability and infectivity of HCV viruses. The relationship between CD81-binding capability and infectivity of the mutant viruses were analyzed by plotting the relative CD81-binding capability of various viral stocks in panel (C) on the x-axis and relative viral titers of the stocks in panel (B) on the y-axis. The correlation co-efficient was calculated using sigma plot.

Viral infectivity of JFH-R m2 was increased about 7-fold when we collected the culture supernatant at 5 days after transfection with 5 days accumulation (Fig. 4B). However, viral infectivity of JFH-R m2 was similar to wild type virus when we collected the culture supernatants at 2 days after transfection with 2 day virus accumulation (Fig. 4C). Based on these findings, we made hypothesis that mutation in E2 protein increased the viability of HCV in culture supernatant at 37°C. To examine the effect of the adaptive mutation in E2 protein on the viability of the extracellular virion in culture media, we incubated the same infectious dose of JFH, JFHm2, JFH-m3 and JFH-m4 viruses at 4°C or 37°C for the times indicated in Figure 5A and 5B, and measured their infectivity. Except for a slight, but insignificant, reduction in the infectivity of viral stocks stored at 4°C for 48 hours, there was no significant difference in infectivity among the four viruses after incubation at 4°C for 48 hours (Fig. 5A). However, differences in virus viability at 4°C among the mutant viruses were observed when viruses were incubated at 4°C up to 3 weeks (Fig. S2B). The half life of the wild type virus was about 2 weeks at 4°C as reported by Ciesek et al. [21]. Viability of the E2 mutant was higher than that of wild type virus even at 4°C (Fig. S2B). The p7 mutation also weakly contributed to the virus viability even though the extent of stabilization was smaller than the E2 mutation (Fig. S2B). The viability of virions decreased dramatically after incubation of viral stocks at 37°C (Fig. 5B). This decrease was greatest for wild-type and JFH-m3 viruses, which both had a half-life of 2.5 to 3 hours at 37°C (Fig. 5B). In contrast, the half-life of JFH-m2 and JFH-m4 viruses was 5 to 6 hours at 37°C (Fig. 5B), suggesting that the mutation in the E2 protein generated by the culture system increases virion viability at 37°C. Interestingly, the viability of infectious JFH virus without the adaptive E2 mutation in the cell culture system (2.5 to 3 hours) was similar to that of HCV particles in infected patients (∼3 hours) [22], [23].

In order to understand the molecular basis of the increased viability of the E2 mutant virus at 37°C, we investigated whether the E2 mutant has acquired prolonged capability of interaction with the best known HCV receptor protein CD81 at 37°C. For this purpose, we generated a recombinant fusion protein (CD81-Fc) containing the Fc region of human IgG and the large extracellular loop (LEL) of human CD81 that is responsible for the interaction with HCV E2 [24]. After incubation of the same infectious dose of JFH, JFH-m2, JFH-m3, and JFH-m4 viruses at 37°C for the times indicated in Figure 5C, virion particles were precipitated by a protein G agarose resin conjugated with the CD81-Fc fusion protein (CD81-Fc-conjugated resin). The amounts of viral RNAs precipitated by the CD81-Fc-conjugated resin were determined by quantitative RT-PCR. The ratios of bound RNAs to total RNAs were approximately the same among the wild type and mutant viruses before incubation at 37°C (Fig. S3). The virion particles associated with the CD81-Fc-conjugated resin and the HCV RNA entered into Huh7.5.1 after infection was drastically reduced by the incubation of virus stocks at 37°C (Fig. 5C and Fig. S4). This indicates that the amounts of virions which can interact with the viral receptor CD81 decrease dramatically by the incubation of the viral stock stocks at 37°C. In other words, the E2 proteins on the virus envelopes are inactivated very rapidly at 37°C. The reducing rate of the resin-bound virions at 37°C differs greatly among the mutant viruses. The viruses with the mutation in the E2 protein (JFH-m2 and JFH-m4) showed higher sustainment of CD81-binding capability at 37°C than the viruses containing wild-type E2 (JFH and JFH-m3) (Fig. 5C). We analyzed the relationship between the CD81-binding capability of viruses and the infectivity of the viruses Fig. 5D). A linear correlation was observed between the CD81-binding capability and the infectivity of viruses (R^2^ = 0.8834). The linear relationship between the CD81-binding capability and the virus infectivity was also observed from individual wild-type and mutant viruses (Fig. S5). The data collectively indicate that the E2 protein is unstable at the physiological temperature, and incubation of viruses at 37°C results in loss of the CD81-binding capability of E2. The mutation in the E2 prolongs the CD81-binding capability of E2 protein resulting in higher infectivity of the mutant viruses JFH-m2 and JFH-m4. It is noteworthy that the mutation in the p7 also partially increases viability of virion particles at 37°C even though the effect is not as strong as the E2 mutation (Fig. 5).

**Figure 6:**
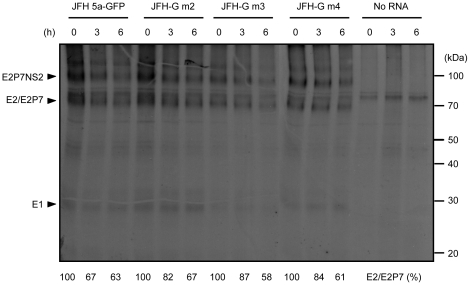
Processing of the E2-P7-NS2 polypeptides of JFH 5a-GFP, JFH-G m2, JFH-G m3 and JFH-G m4 viruses. Huh7.5.1 cells were transfected with the indicated RNAs and then incubated for 48 hours. Newly synthesized proteins were labeled with [^35^S]methionine/cysteine for 24 hours and then chased with unlabeled media for 0, 3 or 6 hours. Mock-transfected cells were used as a negative control. The labeled cell lysates were immunoprecipitated using an E2 specific antibody (AP33). Immunocomplexes were resolved by SDS-PAGE and E2-related proteins were visualized by autoradiography. HCV proteins are indicated by arrowheads on the left, and the positions of the molecular weight markers are given on the right. The relative amounts of E2/E2p7, which were normalized to those in starting time point (t_0_), are depicted below the figure.

To examine the possibility that the adaptive mutations in E2 and p7 influence the level and processing of the E2-p7-NS2, we pulse-labeled proteins in Huh7.5.1 cells transfected with JFH 5a-GFP, JFH-G m2, JFH-G m3, or JFH-G m4 RNAs. Immunoprecipitation of viral proteins with an E2-specific monoclonal antibody (AP33) revealed no significant differences in the amounts of mature E2/E2p7 protein or unprocessed E2-p7-NS2 protein among the various mutants (Fig. 6). Therefore, the adaptive mutations do not affect the level or processing of E2, p7 or NS2 proteins.

In order to test the usefulness of the cell culture-adapted virus in developing anti-viral agents against HCV, we tested effects of anti-HCV agents [interferon-alpha and BILN2061 (a protease inhibitor)] on wild type HCV (JFH 5a-Rluc) and the mutant virus (JFH-R m4) containing the E2/p7 double mutation (Fig. S6). Proliferation of the mutant virus (JFH-R m4) decreased dramatically in dose dependent manners by the anti-HCV agent treatments similarly to JFH 5a-Rluc containing wild type E2 and p7 proteins (Fig. S6). This clearly demonstrates that the virus with the adaptive mutations (JFH-R m4) is useful for screening and testing anti-HCV effects of various agents.

## Discussion

In this study, we developed an efficient HCV cultivation system by selecting for HCVs with cell culture-adaptive mutations. The adaptive mutations increased the infectious viral titer by about 100-fold (Fig. 3D) without affecting the expression of viral protein or viral RNA replication (Fig. 3B and 4A). Two mutations, one located in E2 and one in p7, were responsible for enhanced virus infection, with each increasing viral infectivity at different stages of the HCV life cycle.

The T563I mutation located in the E2 protein enhanced virus infectivity by increasing the viability of the virion in culture media at 37°C (Fig. 5B). To our knowledge, this is the first report describing a mutation that increases virion's viability in culture media at 37°C in in-vitro HCV cultivating system. For instance, a G451R mutation in the E2 protein that increased infectivity titer by 10-fold, identified by Zhong *et. al*, has a selective advantage at the entry stage of virus [25], and an N534K mutation in E2 protein identified by Delgrange *et. al*, facilitates the infection of JFH virus [26]. In this latter study, the authors suggested that the higher infectivity of the N534K mutant virus was attributable to a change in the glycosylation status of the E2 protein.

The E2 protein is an envelope protein that is exposed on the surface of a virion particle and recognizes viral receptor protein(s), such as CD81, on the surface of host cells. Interestingly, the mutation in the E2 protein (T563I), which results in prolonged CD81-binding capacity of E2 at 37°C (Fig 5C), is closely located to the CD81-binding site according to the recent paper describing the tertiary organization of HCV E2 ectodomain [27]. Therefore, it is plausible that the E2 mutation may increase the heat stability of the E2 protein by augmenting the maintenance of E2 conformation which allows the binding of CD81. This prolonged binding capability of E2 protein with CD81 results in higher infectivity of the mutant virus at the physiological temperature (Figures 5B and 5C).

The high turnover rates of HCV are very likely contribute to the rapid generation of viral diversity and the viral escape from the host immune surveillance and antiviral therapy. This may be one of the reasons of the frequent development chronic hepatitis by HCV infection. Nevertheless, HCVs with longer half lives are generated in vivo, which were revealed by the observation of various HCVs having different half-lives from HCV patients [22].

Two functions of the p7 protein in HCV proliferation have been suggested based on earlier studies on p7 and other structurally similar viral proteins. P7 is considered to be a member of the viroporins, similar to 6k of alpha-viruses, M2 of influenza virus A and Vpu of HIV-1 [28]. Indeed, the p7 protein has been shown to have ion channel activity in artificial lipid membranes [12], [29], [30], [31]. One untested possibility is that the p7 N765D mutation changes the activity of the ion channel, resulting in increased infectivity of the virus. Alternatively, p7 may facilitate virus production by interacting with other viral proteins, similar to the M2 protein of influenza virus, which facilitates virus production through an interaction with M1 [32]. Previous studies have reported that p7 interacts with other viral proteins and suggested that these interactions are essential for virus production [14], [33], [34]. For instance, p7 has been shown to play an important role in virus assembly [35]. In this context, a mutation in the p7 protein has been reported to compensate for virus assembly defects that arise due to mutations in the core protein [34]. Moreover, using an intergenotypic chimeric HCV cultivation system, it has been shown that two compensatory mutations in p7 and NS2 proteins play a key role in the assembly and release of infectious virus, and that the interaction between p7 and NS2 proteins are essential for this process [33].

A number of observations reported here also support the conclusion that the N765D mutation in the p7 protein increases virus production by enhancing viral assembly and/or morphogenesis steps. First, time-dependent replication levels are very similar among wild type and m2, m3 and m4 mutations (Fig. 4A). Second, the time required for virus release from viral infection is shorter in m3 and m4 mutants (Fig. 4C). Finally, the ratio of infectious virion particles in the media to that inside the cells is similar among wild type and m2, m3 and m4 mutations (Fig. 4E). Therefore, the N765D mutation may augment virus production by modulating the interaction(s) with other viral proteins involved in virion production, such as the core and NS2. This N765D mutation has been observed in other JFH variants adapted to Huh7.5.1 cells. For instance, Kaul and co-workers found that the same mutation in p7 was included among the cell culture-adapted mutations that increased infectivity of HCV [14], [15]. Recently, Russell *et. al* also suggested that N765D facilitates viral assembly and/or morphogenesis [35].

Interestingly, different patterns of virus distribution were observed in cells infected by JFH-G m2 and JFH-G m3 viruses (panel d and e in Fig. 3C). The JFH-G m2 virus containing a mutation in the E2 protein showed a widely dispersed infection pattern, with the presence of many foci. In contrast, the JFH-G m3 virus containing a mutation in the p7 protein was distributed in a sporadic but focused infection pattern; these cells had fewer foci, and a number of cells had a single focus of infection. This difference may be attributable to the characteristics of the individual mutations. The JFH-G m2 virus, containing a mutation in the E2 protein that increased viability, may be able to travel over greater distances than wild-type virus before it is inactivated, resulting in a more dispersed infection pattern. JFH-G m3 virus, which has a mutation in p7 that facilitates faster virus production, may yield fewer, larger foci. The JFH-G m4 virus containing both mutations acquired these two advantages and showed that maximal infectivity, reflecting a synergistic effect of two mutations.

One mutation in the E1 protein (H316R) had a negative effect on viral infectivity (Fig. 3D). Nakai *et. al* showed that four amino acids (residues 312–315) of the E1 protein are crucial for interacting with the assembled core proteins [36], and four more (residues 316–319) were partially involved. Therefore, it is likely that the H316R mutation hampers virus production by inhibiting the interaction between E1 and assembled HCV core proteins.

Recently, Han et al. found mutations in the nonstructural proteins NS3 and NS5a enhancing the production of HCV containing GFP gene similarly to the JFH 5a-GFP virus [37]. It would be a desirable experiment to test whether the mutations in E2 and p7 described in this paper have a synergistic effect with the mutations in NS3 and NS5a.

In summary, we have developed an efficient HCV cultivation system through continuous culture of JFH containing a reporter (GFP) in NS5A. Viral yield of mutants harboring adaptive mutations selected during culture was approximately 100-fold higher than that of the original JFH 5a-GFP virus. Various reporters, including a luciferase gene, could be incorporated at the GFP site without reducing viral infectivity. This system will be useful for investigating the viral life cycle and developing new anti-HCV drugs and vaccines.

## Materials and Methods

### Plasmid construction

The pJFH 5a-GFP and pJFH 5a-Rluc plasmids have been described previously [20]. A pJFH 5a-GFP plasmid containing adaptive mutations was constructed by amplifying the structural region encoding the core to NS2 using RT-PCR (Fig. 1B). One microgram of total RNA and a primer designated csp 426 (5′-CCG AGA GCA CAC AGC TG-3′) were mixed, heated at 65°C for 10 min, cooled, and then reverse transcribed with Expand RT (Roche Biochemicals) according to manufacturer's instructions. Long-distance PCR was performed using Expand Long template PCR system (Roche Biochemicals) and the primers csp 423 (5′-GCC TAG CCA TGG CGT TAG-3′) and csp 427 (5′-TCG GAA GAG CCC AAC GAC-3′). The amplified DNA fragment was digested with Age I and Avr II and inserted into the parental pJFH 5a-GFP. Of the twelve independent clones obtained, three, designated Ad 9, Ad 12 and Ad 16, were found to have RNA transcripts that expressed viral proteins. To construct GFP-containing mutant constructs, Ad 9 was digested with AgeI and BsiWI (pJFH-G m1), BsiWI and KpnI (pJFH-G m4), KpnI and AvrII (pJFH-G m5) or Asc I (pJFH-G m2 and pJFH-G m3), and the resulting fragments were inserted into pJFH 5a-GFP. To construct Rluc-containing mutant variants of m2, m3 and m4 (pJFH-R m2, pJFH-R m3 and pJFH-R m4, respectively), the corresponding pJFH-GFP mutant constructs were digested with AgeI and AvrII and inserted into pJFH 5a-Rluc.

### Antibodies

Antibodies against the core and NS5a used in western blotting were gifts from Ralf Bartenschlager (University of Heidelberg). The anti-core and anti-NS5a antibodies used in immunofluorescence applications were from Affinity Bioreagents and Austral Biologicals, respectively. The antibody against E2 (AP 33) was a gift from Arvind Patel (University of Glasgow) [38]. The antibody against GAPDH was purchased from AbD SeroTec.

#### Cell culture and TCID_50_ assay

Huh7.5.1 cells [1] were grown in Dulbecco's modified Eagle's medium (DMEM; Gibco) supplemented with antibiotics (100 U/ml penicillin; 10 µg/ml streptomycin) and 10% fetal bovine serum (Sigma) at 37°C in a humidified 6.0% CO_2_ environment. The infectivity of luciferase reporter-containing HCV was determined as previously described [20]. The tissue culture 50% infectivity dose (TCID_50_) was calculated based on previously described methods [17]. Briefly, Huh7.5.1 cells were plated into 96 well at 5×10^3^ cells per well. Culture supernatants were serially diluted 10-fold in normal growth media. 8 wells per dilution were infected. 3 days after infection, whole plates were investigated using fluorescence microscope. Wells containing GFP positive cells were counted as positive and TCID_50_ was determined using the number of these positive wells.

### Fluorescence Microscopy

Fluorescence microscopy was performed as described previously [20], [39].

### Luciferase assay

Luciferase assays were performed using a luciferase assay kit (Promega) according to manufacturer's instructions, as described previously [40].

### RNA transcription and RNA electroporation

*In vitro* transcription and RNA electroporation were performed as described previously [18].

### Production of lysates from HCV RNA-transfected cells by freeze-thawing

Cell culture supernatants from JFH RNA-transfected Huh7.5.1 cells were harvested and cells were washed three times with PBS, scraped and centrifuged for 2 min at 1000×g. Cell pellets were resuspended in a volume of DMEM/10% FBS equivalent to the volume of harvested culture supernatants and subjected to four freeze-thaw cycles using liquid nitrogen and a heating block set to 37°C. Lysates were centrifuged at 1000×g for 2 min to remove cell debris and passed through a 0.45 µm filter.

### Metabolic radiolabeling of proteins and immunoprecipitation

Metabolic radiolabeling of proteins and immunoprecipitation were performed as described previously [15].

### Determination of viability of viruses

The titers of JFH, JFH-m2, JFH-m3 and JFH-m4 viruses were determined by counting plaques visualized by a immune-fluorescence method using Huh7.5.1 cells [18]. Viral stocks were serially diluted in DMEM-10% FBS and inoculated on Huh7.5.1 cells. The HCV-infected cells were cultivated for 72 hours, and then plaques were visualized by an antibody against HCV core. The viral titers were measured three times, and mean value and standard deviation are depicted in the graphs.

### CD81 interaction assay

The CD81-Fc fusion protein and IgG-Fc protein were expressed in 293 T cells and then purified by using protein G agarose beads. The purified CD81-Fc proteins (12.5 µg) were conjugated with protein G agarose resin by incubating them together at 4°C for 4 hours. The viral stocks of JFH, JFH-m2, JFH-m3, and JFH-m4 were incubated with CD81-Fc-conjugated resin at 4°C over night. The resins were suspended in 300 µl PBS, and then the resin-bound viral RNAs were extracted using TRIzol LS (Invitrogen). The amounts of resin-bound virions were measured by quantitative RT-PCR using primers HCV RT F(5′-TGA GGA ACT ACT GTC TTC ACG-3′) and HCV RT R(5′-ATC AGG CAG TAC CAC AAG GC-3′).

## Supporting Information

Figure S1 **Mutations in E2 and p7 proteins increased viral infectivity.** Huh7.5.1 cells were infected with JFH, JFH m-2, JFH m-3, or JFH m-4 viruses containing same mutations as JFH 5a-GFP, JFH-G m1, JFH-G m2, JFH-G m3, JFH-G m4 or JFH-G m5 viruses, respectively. However, these viruses do not contain a reporter gene (GFP). Viral titers were determined using a TCID_50_ assay. The bars and lines represent the means and standard deviations, respectively, from three independent experiments.
(TIF)

Figure S2 **Thermal effects on viabilities of viruses containing mutations in E2 and p7.** The amounts of infectious viruses remaining in the media after thermal treatments [incubation at 4°C (A and B), 37°C (C), or room temperature (D) for the indicated times] were determined by measuring luciferase activities after infecting Huh-7.5.1 cells with JFH 5a-Rluc, JFH-R m2, JFH-R m3 and JFH-R m4 viruses. The relative viral infectivity at each time point is depicted after normalization to the viral infectivity before the thermal treatments that is set to 1. The bars and lines represent the means and standard deviations, respectively, from three independent experiments.
(TIF)

Figure S3 **Proportion of HCV viruses precipitated by a CD81-Fc-conjugated resin.** The amounts of total viral RNAs from same infectious dose of JFH, JFH-m2, JFH-m3, and JFH-m4 viruses (Total) and viral RNAs bound to a CD81Fc resin (IP with CD81Fc) were measured by quantitative RT-PCR. The copy numbers of HCV RNAs (A) and the ratios of bound to total viral RNAs (B) are depicted. The bars and lines represent the means and standard deviations, respectively, from three independent experiments. The ratios of bound to total RNAs were approximately the same among the wild type and mutant viruses.
(TIF)

Figure S4 **Measurement of virus entry.** (A) The same infectious dose of JFH, JFH-m2, JFH-m3, and JFH-m4 viruses were incubated at 37°C for the indicated times, and then incubated with Huh7.5.1 cells for 3 hours. The HCV-infected cells were washed five times with PBS, and RNAs in the cells were isolated. The amounts of viral RNAs in the cells were measured by quantitative RT-PCR. The relative amounts of viral RNAs are depicted. The dots and lines represent the means and standard deviations, respectively, from three independent experiments. (B) The relationship between CD81-binding capability and the entry of viruses. The relationship between CD81-binding capability and the entry of viruses was analyzed by plotting the relative CD81-binding capability of viruses in Figure 5C on the x-axis and relative entry of the viruses in panel (A) on the y-axis. The correlation co-efficient was calculated using sigma plot.
(TIF)

Figure S5 **The relationship between CD81-binding capability and infectivity of individual HCV virus.** The relationship between CD81-binding capability and infectivity of wild-type or individual mutant virus was analyzed by plotting the relative CD81-binding capability of a specific viral stock in Figure 5C on the x-axis and relative viral titer of the stock in Figure 5B on the y-axis. The correlation co-efficient was calculated using sigma plot.
(TIF)

Figure S6 **The effects of antiviral agents on cell culture-adapted virus.** Huh7.5.1 cells were infected with JFH 5a-Rluc virus or JFH-R m4 and then treated with the indicated concentration of BILN 2061 (A) or interferon-alpha (B) for 3 days. Cells were harvested, and luciferase activities in the cells reflecting the amounts of viruses were measured at 3 days post infection. The relative amounts of viruses are depicted by setting the luciferase activity in mock-treated cells to 1. The dots and lines represent the means and standard deviations, respectively, from three independent experiments.
(TIF)
