# Supplementary figures and images for: Generation of a Cell Culture-Adapted Hepatitis C Virus with Longer Half Life at Physiological Temperature
Source: PLoS One. 2011 Aug 4;6(8):e22808. doi: 10.1371/journal.pone.0022808 (PMC3150383; doi:10.1371/journal.pone.0022808)

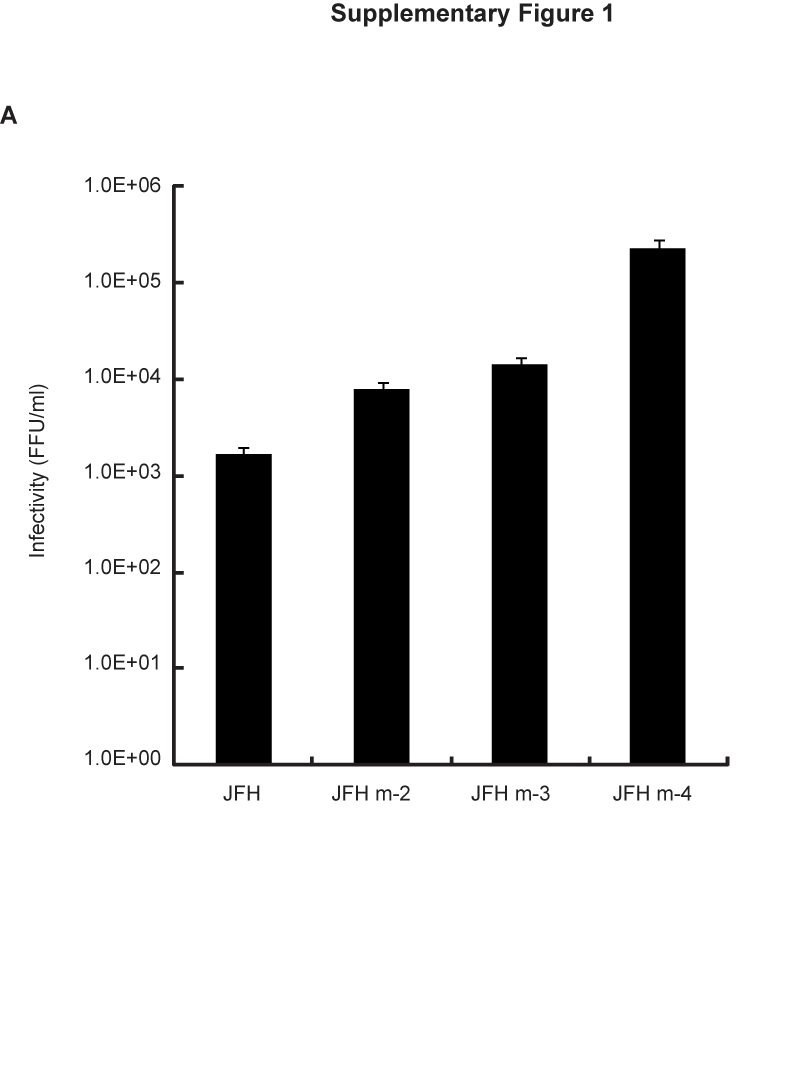

Supplement: Figure S1 — Mutations in E2 and p7 proteins increased viral infectivity. Huh7.5.1 cells were infected with JFH, JFH m-2, JFH m-3, or JFH m-4 viruses containing same mutations as JFH 5a-GFP, JFH-G m1, JFH-G m2, JFH-G m3, JFH-G m4 or JFH-G m5 viruses, respectively. However, these viruses do not contain a reporter gene (GFP). Viral titers were determined using a TCID50 assay. The bars and lines represent the means and standard deviations, respectively, from three independent experiments. (TIF) [file pone.0022808.s001.tif]

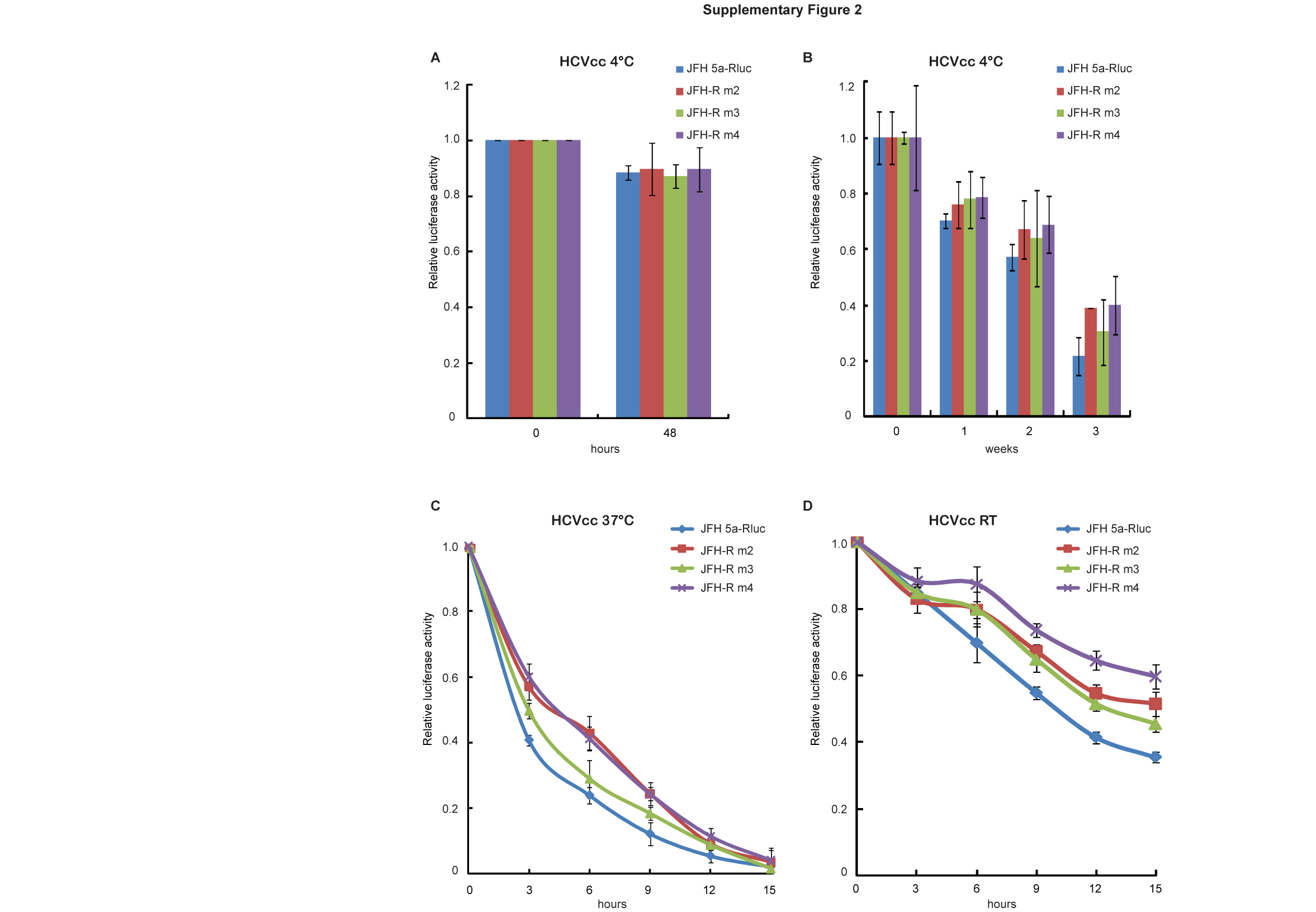

Supplement: Figure S2 — Thermal effects on viabilities of viruses containing mutations in E2 and p7. The amounts of infectious viruses remaining in the media after thermal treatments [incubation at 4°C (A and B), 37°C (C), or room temperature (D) for the indicated times] were determined by measuring luciferase activities after infecting Huh-7.5.1 cells with JFH 5a-Rluc, JFH-R m2, JFH-R m3 and JFH-R m4 viruses. The relative viral infectivity at each time point is depicted after normalization to the viral infectivity before the thermal treatments that is set to 1. The bars and lines represent the means and standard deviations, respectively, from three independent experiments. (TIF) [file pone.0022808.s002.tif]

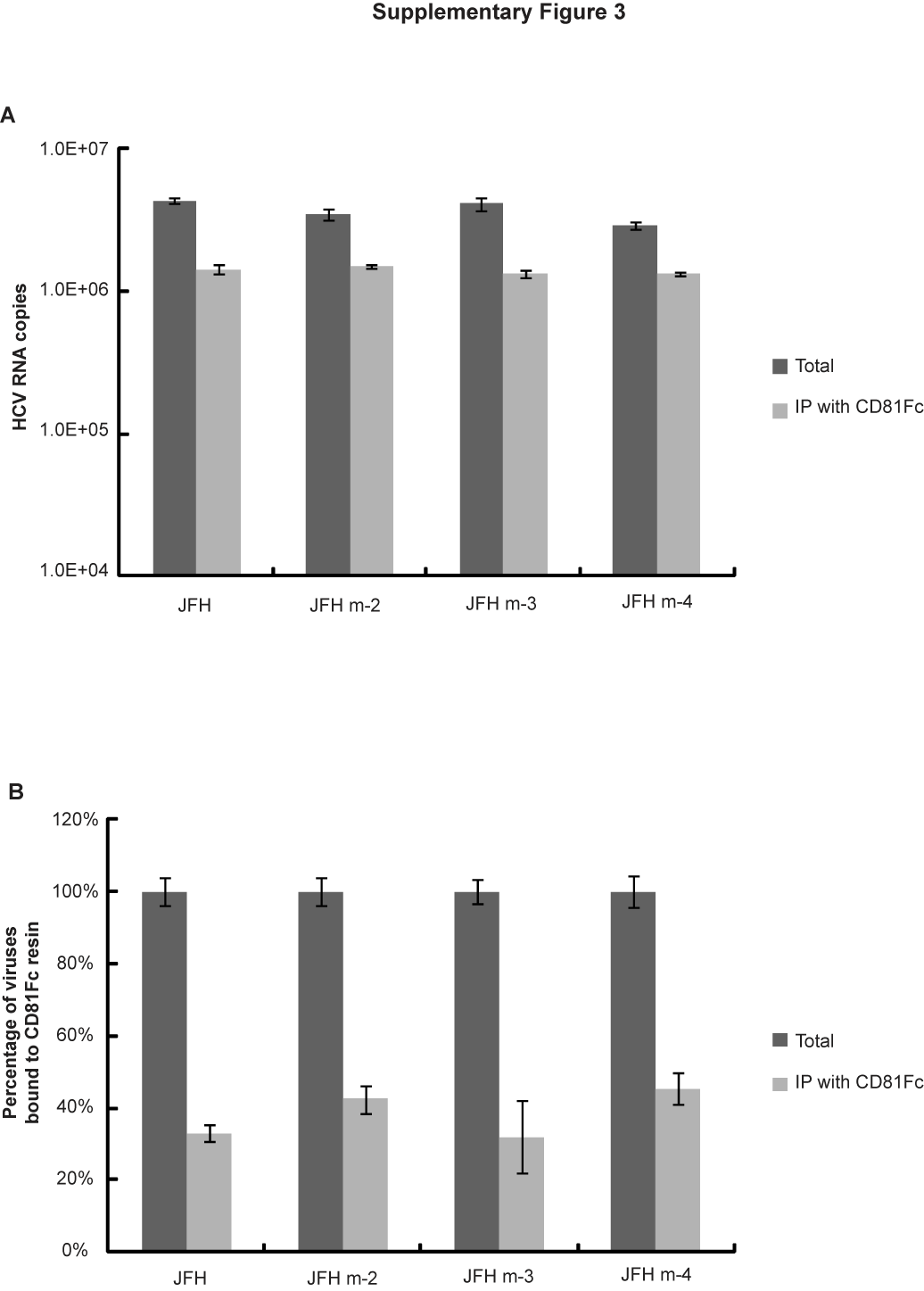

Supplement: Figure S3 — Proportion of HCV viruses precipitated by a CD81-Fc-conjugated resin. The amounts of total viral RNAs from same infectious dose of JFH, JFH-m2, JFH-m3, and JFH-m4 viruses (Total) and viral RNAs bound to a CD81Fc resin (IP with CD81Fc) were measured by quantitative RT-PCR. The copy numbers of HCV RNAs (A) and the ratios of bound to total viral RNAs (B) are depicted. The bars and lines represent the means and standard deviations, respectively, from three independent experiments. The ratios of bound to total RNAs were approximately the same among the wild type and mutant viruses. (TIF) [file pone.0022808.s003.tif]

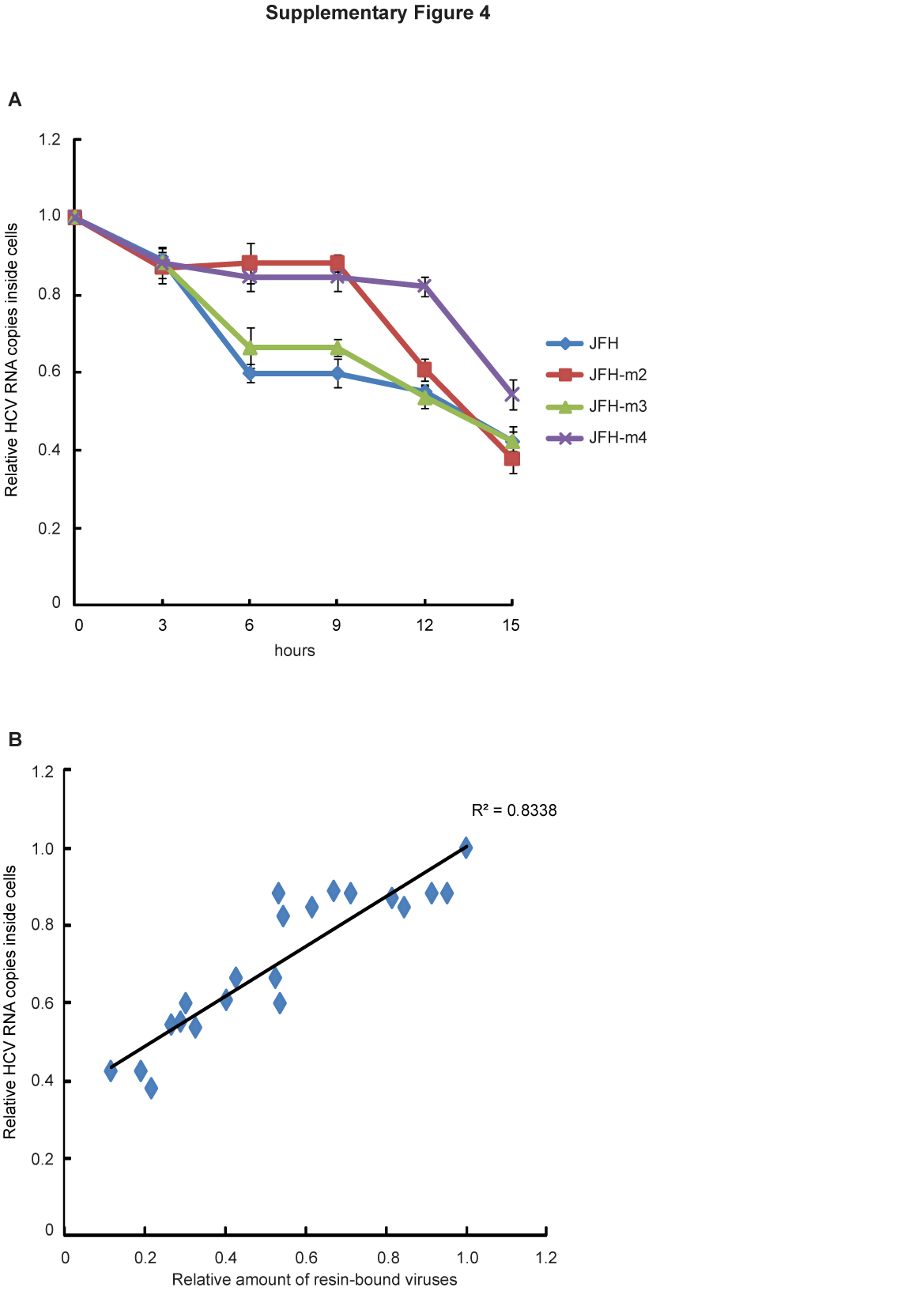

Supplement: Figure S4 — Measurement of virus entry. (A) The same infectious dose of JFH, JFH-m2, JFH-m3, and JFH-m4 viruses were incubated at 37°C for the indicated times, and then incubated with Huh7.5.1 cells for 3 hours. The HCV-infected cells were washed five times with PBS, and RNAs in the cells were isolated. The amounts of viral RNAs in the cells were measured by quantitative RT-PCR. The relative amounts of viral RNAs are depicted. The dots and lines represent the means and standard deviations, respectively, from three independent experiments. (B) The relationship between CD81-binding capability and the entry of viruses. The relationship between CD81-binding capability and the entry of viruses was analyzed by plotting the relative CD81-binding capability of viruses in Figure 5C on the x-axis and relative entry of the viruses in panel (A) on the y-axis. The correlation co-efficient was calculated using sigma plot. (TIF) [file pone.0022808.s004.tif]

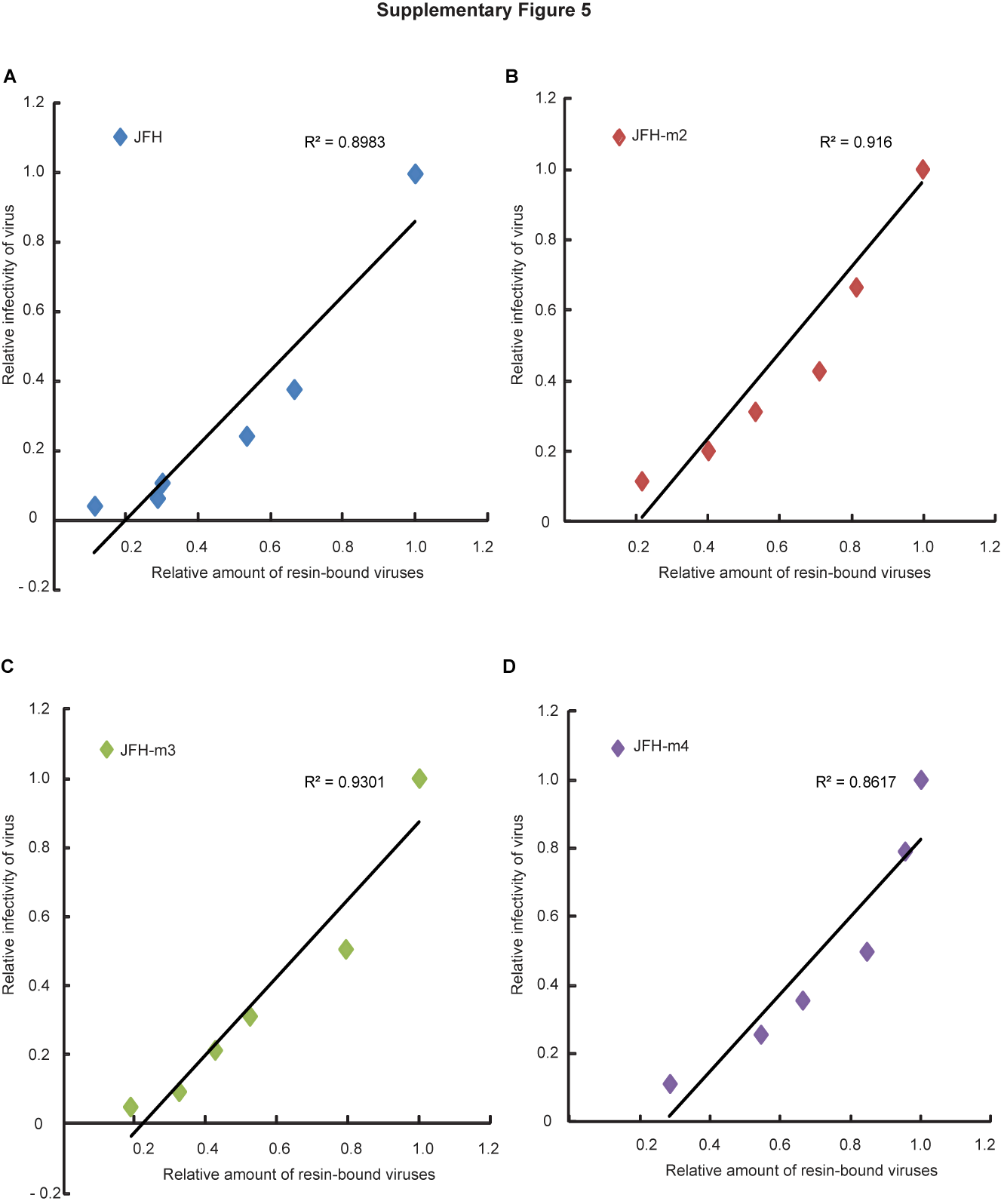

Supplement: Figure S5 — The relationship between CD81-binding capability and infectivity of individual HCV virus. The relationship between CD81-binding capability and infectivity of wild-type or individual mutant virus was analyzed by plotting the relative CD81-binding capability of a specific viral stock in Figure 5C on the x-axis and relative viral titer of the stock in Figure 5B on the y-axis. The correlation co-efficient was calculated using sigma plot. (TIF) [file pone.0022808.s005.tif]

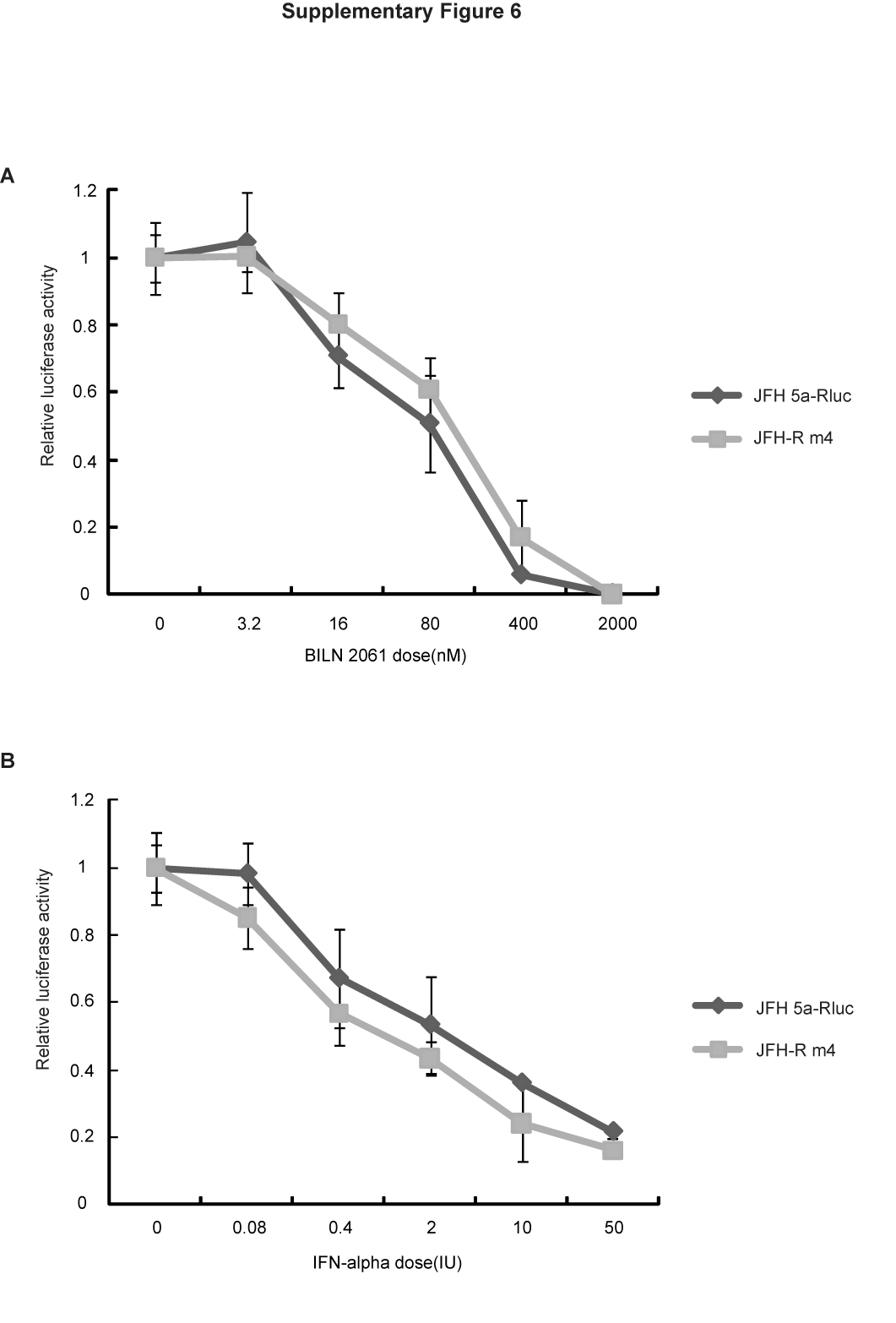

Supplement: Figure S6 — The effects of antiviral agents on cell culture-adapted virus. Huh7.5.1 cells were infected with JFH 5a-Rluc virus or JFH-R m4 and then treated with the indicated concentration of BILN 2061 (A) or interferon-alpha (B) for 3 days. Cells were harvested, and luciferase activities in the cells reflecting the amounts of viruses were measured at 3 days post infection. The relative amounts of viruses are depicted by setting the luciferase activity in mock-treated cells to 1. The dots and lines represent the means and standard deviations, respectively, from three independent experiments. (TIF) [file pone.0022808.s006.tif]
